# Somatostatin receptor-directed molecular imaging for therapeutic decision-making in patients with medullary thyroid carcinoma

**DOI:** 10.1007/s12020-022-03116-6

**Published:** 2022-06-25

**Authors:** Sebastian E. Serfling, Yingjun Zhi, Felix Megerle, Martin Fassnacht, Andreas K. Buck, Constantin Lapa, Rudolf A. Werner

**Affiliations:** 1 Department of Nuclear Medicine, University Hospital Würzburg, Würzburg, Germany; 2 Department of Otorhinolaryngology, Plastic, Aesthetic and Reconstructive Head and Neck Surgery, University Hospital Würzburg, Würzburg, Germany; 3 Division of Endocrinology and Diabetes, Department of Medicine I, University Hospital Würzburg, Würzburg, Germany; 4 Nuclear Medicine, Faculty of Medicine, University of Augsburg, Augsburg, Germany; 5 Johns Hopkins School of Medicine, The Russell H Morgan Department of Radiology and Radiological Science, Division of Nuclear Medicine, Baltimore, MD USA

**Keywords:** Somatostatin receptor, SSTR-PET/CT, Tyrosine kinase inhibitor, Medullary thyroid carcinoma, Theranostics, Peptide receptor radionuclide therapy

## Abstract

**Background:**

Somatostatin receptor (SSTR) positron emission tomography/computed tomography (PET/CT) is increasingly deployed in the diagnostic algorithm of patients affected with medullary thyroid carcinoma (MTC). We aimed to assess the role of SSTR-PET/CT for therapeutic decision making upon restaging.

**Methods:**

23 pretreated MTC patients underwent SSTR-PET/CT and were discussed in our interdisciplinary tumor board. Treatment plans were initiated based on scan results. By comparing the therapeutic regimen before and after the scan, we assessed the impact of molecular imaging on therapy decision. SSTR-PET was also compared to CT portion of the SSTR-PET/CT (as part of hybrid imaging).

**Results:**

SSTR-PET/CT was superior in 9/23 (39.1%) subjects when compared to conventional CT and equivalent in 14/23 (60.9%). Those findings were further corroborated on a lesion-based level with 27/73 (37%) metastases identified only by functional imaging (equivalent to CT in the remaining 46/73 (63%)). Investigating therapeutic decision making, no change in treatment was initiated after PET/CT in 7/23 (30.4%) patients (tyrosine kinase inhibitor (TKI), 4/7 (57.2%); surveillance, 3/7 (42.8%)). Imaging altered therapy in the remaining 16/23 (69.6%). Treatment prior to PET/CT included surgery in 6/16 (37.5%) cases, followed by TKI in 4/16 (25%), active surveillance in 4/16 (25%), and radiation therapy (RTx) in 2/16 (12.5%) subjects. After SSTR-PET/CT, the therapeutic regimen was changed as follows: In the surgery group, 4/6 (66.7%) patients underwent additional surgery, and 1/6 (16.7%) underwent surveillance and TKI, respectively. In the TKI group, 3/4 (75%) individuals received another TKI and the remaining subject (1/4, 25%) underwent peptide receptor radionuclide therapy. In the surveillance group, 3/4 (75%) underwent surgery (1/4, (25%), RTx). In the RTx group, one patient was switched to TKI and another individual was actively monitored (1/2, 50%, respectively). Moreover, in the 16 patients in whom treatment was changed by molecular imaging, control disease rate was achieved in 12/16 (75%) during follow-up.

**Conclusions:**

In patients with MTC, SSTR-PET/CT was superior to CT alone and provided relevant support in therapeutic decision-making in more than two thirds of cases, with most patients being switched to surgical interventions or systemic treatment with TKI. As such, SSTR-PET/CT can guide the referring treating physician towards disease-directed treatment in various clinical scenarios.

## Introduction

As an orphan disease accounting for 1–2% among all thyroid cancers, medullary thyroid carcinoma (MTC) is associated with loco-regional disease in 70% and widespread disease in up to 10% of the cases [1, 2]. As such, various imaging modalities have been explored to assess the current status quo [3–5]. For instance, functional imaging using somatostatin receptor (SSTR) directed positron emission tomography/computed tomography (PET/CT) has proven useful to detect sites of disease, with a tabulated detection rate of more than 86% in selected cases [6]. On a cellular level, such a high diagnostic accuracy can be explained by the increased expression of SSTR on the tumor cell surface, including subtype 2A [7], which is also primarily targeted by the most commonly used radiotracers (1,4,7,10-tetraazacyclododecane-N,N′,N″,N‴-tetraacetic acid-d-Phe(1)-Tyr(3)-octreotide/−octreotate ([^68^Ga-]DOTATOC/ − TATE)) [8]. Of note, as a theranostic agent, quantification of the target by [^68^Ga]-DOTATOC/ − TATE also allows to select individuals for treatment with ß-emitters, e.g., 177Lu or 90Y. As such, the current guideline of the *European Association of Nuclear Medicine* endorses the use of those radiopharmaceuticals for restaging of patients with MTC [4]. However, such a theranostic approach is currently not approved in clinical practice, despite proven effectiveness in end-stage disease [9]. For instance, a recent meta-analysis reported on a biochemical response in more than 37% of the patients [9]. In particular in the setting of treatment for gastroenteropancreatic neuroendocrine tumors, toxicity profile was also low [10].

Similar to image-based diagnostics, the therapeutic armamentarium for recurrent disease has also been further expanded from external beam radiation (RTx) or active surveillance to novel systemic treatment options, including tyrosine kinase inhibitors (TKI), pasireotide combined with everolimus or peptide receptor radionuclide therapy (PRRT) [1, 11–14]. As such, given the increasing use of SSTR-targeted imaging for MTC [4, 13, 15, 16], we aimed to assess the role of this functional imaging modality as a therapeutic decision aid in a real-world scenario.

## Material and methods

### Patient population

23 consecutive patients (11 female) between 32 and 81 years (51.9 ± 13.4 y) with histologically confirmed MTC (20/23 sporadic (87%), 3/23 hereditary (13%) were included (Table 1). At time of SSTR-directed PET/CT, calcitonin was median 1400 pg/ml (range, 26–42700) and carcinoembryonic antigen was median 32 ng/ml (range, 2–2386). Upon referral by the treating physician, all patients underwent SSTR-directed PET/CT due to suspicion of disease progression based on raising serum calcitonin levels. Informed consent was obtained from all subjects involved in the study. As this was a retrospective investigation, the local ethics committee waived the need for further approval (waiver No.: 2021041503).

**Table 1:** Patient’s characteristics.

No.	Sex	Age (y)	Duration of Disease until imaging (mo)	Metastatic sites on SSTR-PET/CT	Disease	Therapy prior to SSTR-PET/CT	Therapy after SSTR-PET/CT	Histo	Change in management	Staging prior to SSTR-PET/CT	Staging based on SSTR-PET/CT	Controlled disease after SSTR-PET/CT?^#^
1	f	54	35	cLN	sporadic	Surgery (TE, ND)	Surgery (cLN)	P	Y	T1bN1bM0	T1bN1bM0	Y
2	m	32	120	c/medLN	sporadic	Surgery (residual TE)	Surgery (c/medLN)	P	Y	T3N1aM0	T3N1bM0	Y
3	f	81	100	RTT	sporadic	Surgery (TE)	Surgery (residual TE)	P	Y	T3N1aM0	T3N1aM0	Y
4	f	46	4	cLN	sporadic	Surgery (TE, ND)	Surgery (cLN)	P	Y	T3N1M0	T3N1M0	Y
5	m	53	1	c/medLN, bone	sporadic	Surgery (c/medLN)	Surveillance		Y	T4aN1bM1	T4aN1bM1	Y
6	f	26	1	bone, liver	sporadic	Surgery (TE)	TKI (cabozantinib)		Y	T1aN0M1	T1aN0M1	Y
7	m	56	123	c/medLN	sporadic	TKI (vandetanib)	TKI (cabozantinib)		Y	T4aN1bM0	T4aN1bM0	N
8	m	51	220	c/med/abdLN, bone	sporadic	TKI (vandetanib)	TKI (selpercatinib)		Y	T4N1bM1	T4N1bM1	Y
9	m	49	120	c/medLN	sporadic	TKI (vandetanib)	TKI (cabozantinib)		Y	T4N1M0	T4N1M0	Y
10	f	38	161	c/medLN, lung	hereditary	TKI (vandetanib)	PRRT		Y	T4N1bM1	T4N1bM1	N
11	f	51	264	liver	sporadic	Surveillance	Surgery (liver)	P	Y	T1aN0M0	T1aN0M1	Y
12	f	59	78	cLN, LR, lung	sporadic	Surveillance	Surgery (cLN, LR)	P	Y	T1bN1aM1	T1bN1bM1	N
13	m	53	26	cLN	sporadic	Surveillance	Surgery (cLN)	P	Y	T3N0Mx	T3N1aM0	Y
14	m	66	8	cLN, liver	hereditary	Surveillance	RTx (liver)		Y	T2N1aM0	T2N1aM1	N
15	f	36	10	Bone	hereditary	RTx (cervical, spine)	TKI (cabozantinib)		Y	T1aN0M1	T1aN0M1	Y
16	m	66	168	cLN, brain	sporadic	RTx (brain)	Surveillance		Y	T4N1Mx	T4N1M1	Y
17	m	51	360	c/medLN	sporadic	TKI (vandetanib)*	TKI (vandetanib)		N	T4N1bM0	T4N1bM0	na
18	m	73	126	Bone, lung	sporadic	TKI (vandetanib)*	TKI (vandetanib)		N	T2aN0M0	T2aN0M1	Y
19	m	47	84	c/medLN, liver, bone	sporadic	TKI (cabozantinib)*	TKI (cabozantinib)		N	T3N1M0	T3N1M1	N
20	f	50	134	cLN, bone, liver, lung	sporadic	TKI (cabozantinib)*	TKI (cabozantinib)		N	T4aN1bM1	T4N1bM1	N
21	f	60	48	Bone	sporadic	Surveillance	Surveillance		N	T3N0M1	T3N0M1	na
22	m	32	12	Bone, liver	sporadic	Surveillance	Surveillance		N	T4N0M1	T4N0M1	Y
23	f	65	165	Bone	sporadic	Surveillance	Surveillance		N	T4aN0M1	T4aN0M1	N

*y* years, *mo* months, *PET* positron emission tomography, *SSTR* somatostatin receptor, *CT* computed tomography, *Histo* histology (derived from PET-guided surgical specimen), *f* female, *LN* lymph node, *TE* thyroidectomy, *ND* neck dissection, *c* cervical, *na* not available. *m* male, *med* mediastinal, *P* positive for MTC on histological assessment, *RTT* remnant thyroid tissue, *TKI* tyrosine kinase inhibitor, *abd* abdominal, *PRRT* peptide receptor radionuclide therapy, *LR* local recurrence, *RTx* external beam radiation.

*In patients that remained under TKI, dosage of the drug remained the same.

^#^based on first follow-up imaging using RECIST criteria in all available patients (with stable disease, partial or complete response defined as controlled disease).

### Imaging procedures

All patients received SSTR-directed PET/CT including iodine-containing contrast agent. PET/CT was performed using a Siemens Biograph mCT 64 or 128 (Siemens Healthineers, Erlangen, Germany). Imaging was performed 60 min after injection of approximately 120 ± 22 MBq [^68^Ga]-DOTATOC or -TATE. A whole-body scan (ranging from the vertex of the skull to the proximal thighs) was performed. PET/CT section thickness was 5 mm. All PET images were reconstructed iteratively as implemented by the manufacturer (Siemens Healthineers, Erlangen, Germany). For further details, please refer to [17].

### Image analysis

Findings on SSTR-PET/CT were also compared to CT alone by an expert reader (S.E.S.). Allowing for direct comparison between both modalities, CT portion of the SSTR-PET/CT (as part of hybrid imaging) was investigated. We performed a patient-centric and lesion-based analysis. For a patient-based analysis, overall image impression was used, while for a lesion-based analysis, every site of disease was recorded. In this regard, lesions were either classified as exclusively identified by CT or SSTR-PET/CT, respectively, or considered equivalent if both modalities revealed identical findings. [^68^Ga]-DOTATOC/-TATE was investigated in the present analysis, as this is a routinely available radiotracer at our institution. Although [^18^F]-dihydroxyphenylalanine ([^18^F]-DOPA) has been reported to be superior relative to [^68^Ga]-DOTATOC or –TATE for imaging of MTC patients in selected clinical scenarios [18], the latter PET agents are more widely available due to an increased use for gastrointestinal neuroendocrine tumors [19], thereby rendering our findings more relevant for a broader community. PET-based quantification was also conducted by manually segmenting sites of disease using a dedicated software package (syngo.via, Siemens Healthineers, Erlangen, Germany), providing averaged peak standardized uptake value (SUV_peak_) and tumor volume (TV) from PET.

### Investigating the potential of PET signal as an image biomarker

To assess the potential of the PET signal as a PET-based biomarker in the context of MTC, calcitonin was also correlated with PET-derived TV and SUV_peak_.

### Assessment of change in treatment

After having conducted SSTR-directed imaging, all patients were discussed in our interdisciplinary tumor board (including endocrine surgeons, radiation oncologists, and pathologists) and imaging results were presented by an imaging team, consisting of a board-certified nuclear medicine physician and radiologist. Recommendations for change (or no change) in prior therapeutic management was then based on PET findings. Treatment immediately prior to and after SSTR-targeted PET/CT was retrieved from medical health records, thereby allowing to assess treatment changes. In this regard, the referring treating physician always followed recommendations of the tumor board.

### Assessment of impact of management change

We also aimed to determine whether management change had also an impact on outcome. As such, first image-based follow-up after SSTR-directed imaging was also investigated by applying RECIST criteria [20]. Stable disease, partial or complete response were defined as controlled disease [20].

### Statistical analysis

We used GraphPad Prism Software (9.3.1; La Jolla, CA, USA). Descriptive statistics are presented as mean ± SD or median. Correlative analysis was conducted by using Pearson’s correlation. An outlier correction using the ROUT method was also performed. A *p*-value of less than 0.05 was considered significant.

## Results

### In-vivo SSTR expression, but not PET-based TV correlates with calcitonin

SUV_peak_ showed a moderate correlation with calcitonin (*r* = 0.51, *p* = 0.03) at time of imaging, while PET-based TV failed to reach significance (*r* = 0.29, *P* = 0.24).

### SSTR-PET/CT is superior to CT

On a patient-based level, SSTR-PET/CT revealed superior findings in 9/23 (39.1%) subjects when compared to conventional CT (equivalent, 14/23 (60.9%); CT alone, none). In a lesion-based analysis, a total of 73 lesions were detected, with 27/73 (37%) identified exclusively by SSTR-PET/CT. In the remaining 46/73 (63%) metastases, CT and SSTR-PET/CT revealed equivalent findings (superiority of CT in none of the cases). Overall, the following sites of disease were identified by SSTR-PET/CT: 15/23 (65.2%) subjects had lymph node and 4/23 (17.4%) lung involvement, osseous disease was encountered in 10/23 (43.5%) individuals, hepatic lesions in 6/23 (26.1%) and brain metastasis, local recurrence and remnant thyroid tissue in 1/23 (4.3%), respectively.

### SSTR-PET/CT triggers surgery or systemic treatment

**Fig. 1:**
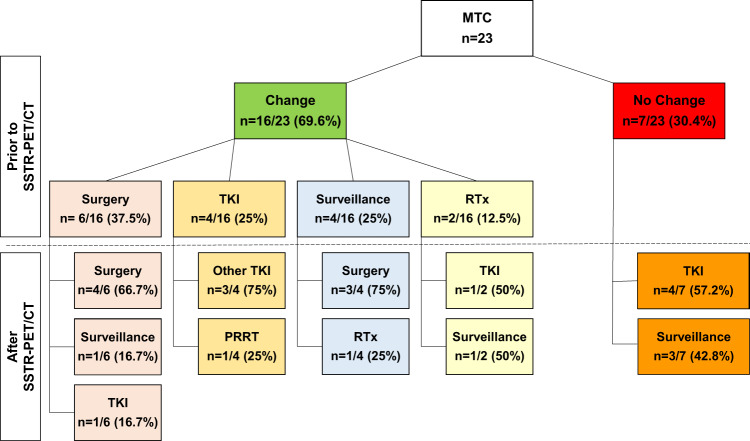
Overview of patients affected with medullary thyroid carcinoma (MTC), which underwent somatostatin receptor (SSTR)-directed positron emission tomography/computed tomography (PET/CT). Therapy prior to and after SSTR-PET/CT is displayed. In more than 69% of the patients, SSTR-PET/CT resulted in treatment initiation, with most patients switched to surgery or tyrosine kinase inhibitors (TKI). PRRT Peptide Receptor Radionuclide Therapy, RTx external beam radiation.

**Fig. 2:**
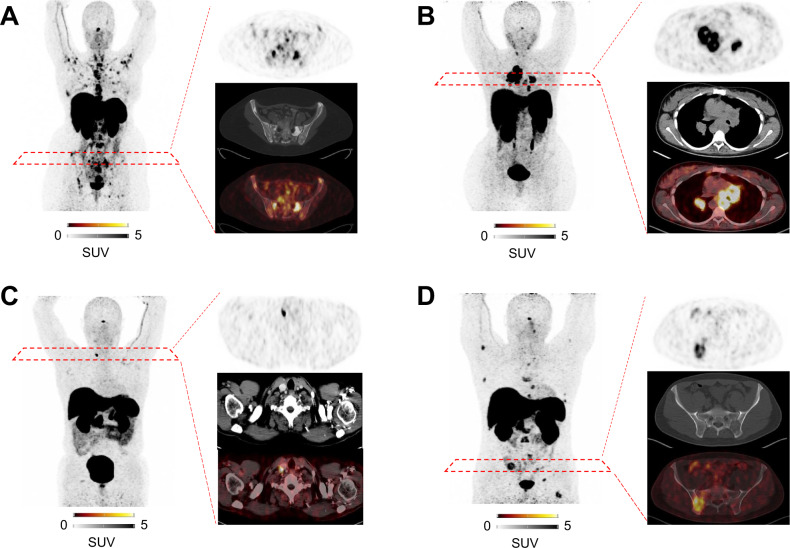
Cases with changes of therapy triggered by somatostatin receptor positron emission tomography/computed tomography (PET/CT). Maximum intensity projection, transaxial PET, CT and PET/CT are displayed. **A** Switch from surgery to tyrosine kinase inhibitor (TKI). After thyroidectomy, somatostatin receptor (SSTR)-directed imaging revealed widespread osseous disease, which triggered systemic treatment with TKI (Case #6, Table 1). **B** Switch from TKI to peptide receptor radionuclide therapy (PRRT). SSTR imaging revealed multiple lung and mediastinal lymph node metastases under TKI with increased uptake and thus, PRRT was initiated (Case #10, Table 1). **C** Switch from surveillance to surgery. Patient was under surveillance. On follow-up SSTR PET/CT, a single cervical lymph node lesion was detected. Guided by the imaging results, the patient underwent surgery (Case #13, Table 1). **D** Switch from external beam radiation (RTx) to tyrosine kinase inhibitor (TKI). After RTx to the spine and cervical region, multiple bone metastases were revealed on SSTR directed imaging, and thus, treatment with TKI was initiated (Case #15, Table 1).

In 7/23 (30.4%) subjects, therapy remained unchanged after SSTR PET/CT, with 4/7 (57.2%) patients remaining under TKI treatment and 3/7 (42.8%) under surveillance, respectively. Imaging, however, triggered a therapeutic decision in the remaining 16/23 individuals (69.6%; Fig. 1; Table 1). Treatment prior to PET included surgery in 6/16 (37.5%), followed by tyrosine kinase inhibitor (TKI) in 4/16 (25%, including vandetanib), active surveillance in 4/16 (25%), and RTx in 2/16 (12.5%) patients, respectively. After SSTR-PET/CT, the therapeutic regimen was altered as follows: In the (former) surgery group, 4/6 (66.7%) subjects underwent additional surgery, and each 1/6 (16.7%) were allocated for active surveillance and TKI (cabozantinib), respectively (Fig. 2A). In the TKI group, 3/4 (75%) patients received another TKI (cabozantinib, selpercatinib) and the remaining subject (1/4, 25%) underwent PRRT (Fig. 2B). In the surveillance group, 3/4 (75%) individuals underwent surgery (Fig. 2C), while the remaining subject (1/4, 25%) was scheduled for RTx. In the RTx group, one patient was switched to TKI (cabozantinib, Fig. 2D) and another individual was actively monitored (1/2 (50%), respectively). Surgical specimens derived from SSTR-PET guided surgery were positive for MTC in 7/7 (100%).

### Management change triggered by SSTR-PET/CT can control disease during follow-up

In 2/23 (8.7%), no follow-up imaging was available. The remaining 21/23 (91.3%) patients had a median follow-up of 5.7 months until subsequent imaging follow-up. In those patients, disease control was achieved in 14/21 (66.7%).

In the 16 patients in whom treatment was changed based on molecular imaging, control disease rate was 12/16 (75%). In patients without change, follow-up was only available in 5/7 (71.4%) and disease sites could be controlled in 2 out of 5 subjects (40%; Table 1).

## Discussion

Investigating 23 subjects affected with MTC who underwent restaging using SSTR-directed PET/CT, the latter imaging modality was superior to CT alone on a patient- and lesion-based level. In addition, molecular imaging led to a change in the therapeutic algorithm in more than two thirds of the cases. In most subjects, functional read-out of SSTR expression triggered either surgical interventions or TKI treatment. Those findings support the notion that SSTR PET/CT can identify both residual disease activity (leading to surgery), but also widespread involvement (prompting systemic treatment with TKI), thereby guiding the referring physician to choose the appropriate treatment upon restaging. In addition, implemented management changes triggered by PET also led to disease control in 75% on follow-up, further supporting the notion of an added clinical value to conduct SSTR-PET/CT in patients with MTC even for outcome.

In patients with MTC, imaging modalities comprise magnetic resonance imaging (MRI), CT, or functional imaging including SSTR-directed PET, 2-deoxy-2-[^18^F]fluoro-D-glucose ([^18^F]FDG), or [^18^F]-DOPA) [4, 21]. In a triple tracer approach investigating all of these radiotracers in 18 MTC patients with recurrent disease, a change in management was recorded in 8/18 (44.4%) of the cases, which was lower when compared to the present study (>69%) [3]. As a possible explanation, all our patients underwent imaging with SSTR-targeting radiotracers instead of administering multiple tracers to the identical patient [3]. Such an approach, however, may cause a bias in scan interpretation, e.g., due to discordant findings on different PETs [3]. In addition, [^18^F]-DOPA demonstrated a higher diagnostic accuracy when compared to [^68^Ga]-somatostatin analogue PET for lesion detection [3]. Thus, in the previous study, novel therapy was mainly initiated by findings on [^18^F]-DOPA, but not SSTR-directed PET/CT [3]. Nonetheless, given the increasing use of SSTR-PET in the context of imaging for neuroendocrine tumors of the gastrointestinal tract [22], [^68^Ga]-labeled somatostatin analogues are virtually available at almost every PET center, in particular as synthesis does not require a costly on-site cyclotron when compared to [^18^F]-DOPA [4]. Thus, reflecting a real-world scenario with a more widespread availability of [^68^Ga]-DOTATATE/-TOC, the herein presented findings indicate that SSTR-targeted molecular imaging may serve as a relevant decision-aid for the practitioner. Further corroborating the clinical utility of SSTR-directed PET, pathology of all patients, which underwent PET-guided surgery, were positive for MTC. Nonetheless, future studies may also investigate the impact on change in the therapeutic algorithm based on other, more sensitive positron-emitting agents, such as [^18^F]-DOPA, preferably in the context of PET-piloted surgical interventions [3].

In the present study, changes of treatment were exclusively based on PET findings, but this advanced imaging modality is not always available at every site treating patients with MTC. Tumor markers, such as calcitonin, however, are easily obtainable and thus, are also routinely assessed during follow-up [1]. As such, future studies may also conduct a physician-based survey, which will then further allow to disentangle the impact of tumor marker fluctuations or imaging on change in management or as a therapeutic decision making tool [23].

MRI in combination with bone scintigraphy has been reported to provide complementary information in patients with skeletal lesions [5]. Nonetheless, no conventional or functional imaging modality alone is sufficient to indicate all needed information on the current *status quo* in patients with MTC [1] and thus, future studies may also include various diagnostic procedures, such as MRI, ultrasound, bone scintigraphy and SSTR-PET/CT. Beyond staging, however, the latter technique also allows to identify patients who are suitable for PRRT [13]. Of note, in our cohort, only one patient was treated in such a theranostic context (Fig. 2B), which is in line with a previous study also reporting on a relatively low number of patients being eligible for PRRT based on imaging with 68Ga-labeled somatostatin analogues [24]. However, given the encouraging survival rates of up to 36–63 months [13, 24], SSTR-PET/CT may still be a useful tool to pave the way for treatment with “hot” somatostatin analogues, in particular in end-stage disease [13].

This study has several limitations. First, despite enrolling one of the largest cohorts imaged with SSTR-PET/CT for restaging in the context of MTC, more patients should be included in future, prospective investigations. In addition, we demonstrated superiority of SSTR-PET/CT on a patient- and lesion-based level in up to 39% of the cases when compared to CT, but an impact of CT alone on therapeutic decision-making cannot be definitely ruled out. In this regard, previous investigations have also reported that conventional imaging techniques (including MRI, bone scans, CT and ultrasound) are superior to SSTR-PET/CT in only 13% of the cases [25]. Nonetheless, future studies should also include more easily available imaging techniques such as ultrasound, as lymph node involvement in the neck is common in those patients [26]. Tumor markers were substantially elevated in our cohort; this, however, may reflect a real-world scenario where patients for evaluation of systemic therapy often present with high calcitonin levels at time of imaging. Scan results provided decision support in a relevant number of subjects, and change in management led to disease control in 75%. However, further analysis should link those PET/CT-based treatment changes to other outcome variables, e.g., progression-free and overall survival.

## Conclusions

Based on SSTR-PET/CT, treatment was initiated in more than two thirds of patients affected with MTC, thereby indicating that this functional imaging modality may provide relevant decision support in the treatment algorithm of those patients. PET triggered either (locoregional or LN) surgery or systemic treatment with TKI, supporting the notion that a functional read-out of SSTR expression can guide the referring treating physician towards the appropriate treatment in various clinical scenarios.
